# Racial and Ethnic Differences in the Perceptions of Health, Work Environment and Experiences of Work-Related Symptoms Among Cleaning Workers

**DOI:** 10.1007/s10903-022-01328-6

**Published:** 2022-01-25

**Authors:** Minjung Kyung, Nicole Collman, Sandra Domeracki, OiSaeng Hong, Soo-Jeong Lee

**Affiliations:** Department of Community Health Systems, School of Nursing, University of California, San Francisco, 2 Koret Way, N505, Box 0608, San Francisco, CA 94143 USA

**Keywords:** Racial and ethnic difference, Health perception, Psychosocial work environment, Work-related symptoms, Reporting, Cleaner

## Abstract

This study explored racial and ethnic differences in perception of work environment, safe work practices, general health status, experience of work-related injury or illness and subsequent symptom reporting and health care seeking behaviors among cleaning workers. This study analyzed cross-sectional data obtained from 183 cleaning workers employed in a university hospital or a health sciences campus in Northern California. The sample included 120 Asians (65.6%), 37 Hispanics (20.2%), and 27 other ethnicities (14.2%); 85.7% were foreign-born. Asian workers perceived lower job control and supervisor support and higher job strain than other workers. The odds of perceiving general health as excellent or very good were lower among Asians compared to Hispanics and Others. Asians who experienced chemical-related symptoms were less likely than Hispanics and others to report the symptoms to their supervisor or seek healthcare. Our study findings indicated racial/ethnic differences in perceptions of work and general health, seeking healthcare, and reporting behaviors among cleaning workers. Asian workers, specifically, may need special attention to improve their experiences of work environments and health in the workplace.

## Background

Cleaning work involves frequent contacts with chemical substances, biological agents, and labor-intensive tasks [1–5]. Previous studies have shown that cleaning workers have a greater risk for musculoskeletal [1, 6, 7], dermal [4, 8, 9], and respiratory health related problems [4, 10–12]. Furthermore, cleaning work is one of the lowest compensated jobs with many psychosocial hazards such as precarious employment conditions, high job demands, low job decision latitude, and low social support, all of which can lead to adverse health effects [2, 5, 13].

Immigrant and minorities constitute a majority of cleaning workforce in industrialized countries, positions that many native workers have vacated [14, 15]. Additionally, immigrant and minority workers are disproportionately employed in higher risk jobs, resulting in higher rates of work-related illnesses and injuries [16]. Due to cultural differences, language barriers, and limited knowledge of available resources for safety, these minority workers are also less engaged in safe work practices [17, 18]. According to previous studies, racial/ethnic minority workers were more likely to experience work-related injuries or illnesses [19, 20] and more likely to perceive their health as poor or fair than White workers [21, 22].

Reporting occupational injury and illness is crucial in identifying workplace hazards and improving worker safety, but underreporting is a common problem in a wide range of workplaces [23]. Despite the higher prevalence of occupational injuries and poorer health perception, minority workers often hesitate to report work-related symptoms to management [23]. Tucker et al. [24] estimated underreporting of work-related injuries or illnesses to range between 29 and 81%. Minority workers were also shown to be less likely to seek healthcare for their illness. In a study by Hoerster et al. [25], 20.6% of migrant workers visited a healthcare provider during a year, compare to 28.5% of workers who are citizens. Yet, there is very limited research that examines racial and ethnic differences in work-related symptom reporting and healthcare seeking among cleaning workers [26, 27]. In a study of 941 Las Vegas hotel room cleaners by Premji and Krause [28], Hispanics had a significantly higher prevalence of work-related pain than non-Hispanics, but seeking healthcare and symptom reporting to management were not different by racial/ethnic groups. In Green et al.’s study of janitors that mostly consisted of Hispanics, more than half of the participants perceived barriers of reporting an injury to their employer [27].

The purpose of this study was to examine racial and ethnic differences regarding perception of the work environment, safe work practices, general health status, experience of a work-related injury or illness and subsequent symptom reporting, and healthcare-seeking behaviors among cleaning workers. This study’s findings can help identify specific minority groups that may require more attention to address disparities in work-related health and safety behaviors within the work environment.

## Conceptual Framework

**Fig. 1:**
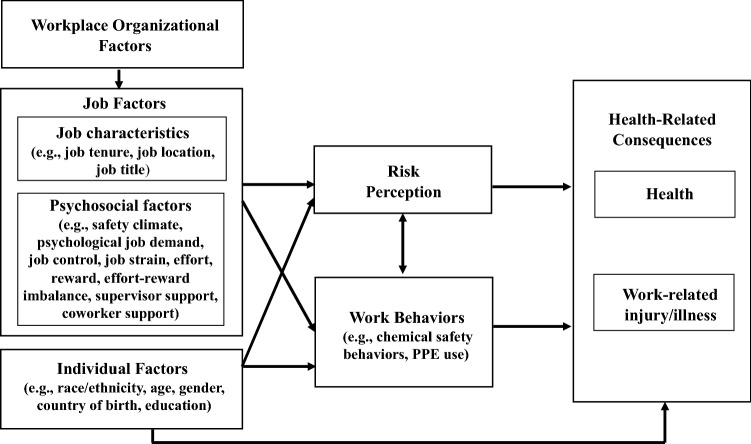
A conceptual framework of the study

Figure 1 shows the conceptual framework of this study, which is modified based on a model by Lee et al. [29]. The framework proposes that workplace organizational factors, psychosocial work factors, job characteristics and individual factors impact workers’ risk perception, their work behaviors, and health-related consequences (i.e., health, work-related injury/illness, health care seeking behaviors, symptom reporting). Although risk perception and work behaviors may mediate or moderate the effects of workplace and job factors, this study examined only their direct effect on health-related consequences.

## Methods

### Study Design and Sample

This cross-sectional study was conducted with a convenience sample of 183 cleaning workers employed in a university medical center and affiliated health sciences campuses in Northern California. Participants included custodians, patient support assistants (PSA), and their supervisors who performed janitorial, cleaning, or housekeeping services and were employed for at least one month. For participant recruitment, the research team provided the information about the study at the department staff meetings and posted study flyers on department bulletin boards and in staff lounges. Since many cleaning staff speak and/or read in Chinese or Spanish, all study information was made available in these languages as well as English. The study was approved by the Institutional Review Board at the University of California, San Francisco.

### Data Collection

Trained bilingual investigators interviewed participants face-to-face using a questionnaire developed in English, Chinese, and Spanish. Participants chose their preferred language. Self-administration of the questionnaire was also used to facilitate participation in the study. All participants signed an informed consent before completing the study questionnaire and received a $25 gift card incentive after completing the questionnaire. The detailed information on the methods can be found in a previous publication [4].

### Variables

#### Demographic and Job Characteristics

Demographic variables included race/ethnicity, age, gender, country of birth (United States [U.S.] or other), and education. Job-related variables included job tenure, job location (hospital or campus), job title (PSA, custodian, or supervisor) and work status (full-time or part-time).

#### Psychosocial Factors

Job demand (5 items), job control (9 items), supervisor support (4 items), and coworker support (4 items) were measured by the Job Content Questionnaire (JCQ) [30]. Job demand refers to a psychological workload associated with work amount, intensity, and speed [13]. Job control indicates the working individual’s potential control over tasks including skill discretion and decision authority [13]. Job strain was calculated as a ratio of job demand (range 12–48) to job control (range 24–96), and a correction factor of 2 was multiplied to account for the difference in scoring between the two scales. As a widely used job stress measure, the JCQ psychometric properties have been previously well described [30]. Effort (6items) and reward (11items) were measured by Effort-Reward Imbalance (ERI) Questionnaire [31] and the Effort-reward ratio was calculated by dividing effort by reward and multiplying by a correction factor of 0.5454. The ERI questionnaire has also been validated by previous studies [31–34]. Safety climate (16 items) was measured using the instrument developed by Zohar and colleague [35], which has demonstrated excellent reliability and good predictive validity. Safety climate refers to workers’ perceptions of workplace safety regarding organizational commitment to safety, communication and feedback, and safety programs, policy and practice [36]. Risk perception (25 items) was assessed by asking workers about their concerns related to their health and chemical exposures from cleaning products or job tasks. Examples of cleaning tasks or products included mixing or diluting cleaning solutions, dusting/sweeping/vacuuming, mopping/wet cleaning/damp wiping, stripping floors, cleaning in-patient rooms, washing patient beds or surgical tables, and cleaning tasks using sprays. The risk perception score was calculated as the mean score of item responses because some items were not applicable depending on one’s job location or job title.

#### Safe Work Practices

To assess safe work practices, participants were asked about chemical safety behavior and use of personal protective equipment (PPE). Chemical safety behavior (8 items) was assessed by using an 8-item questionnaire developed by Lee et al. [4] and participants were asked to indicate the frequency of their engagement in the behaviors. PPE use was measured by asking respondents how often in the past 30 days they wore gloves, safety glasses or goggles, long-sleeved clothing or a rubber apron, face shield, and surgical mask while handling chemicals. All safe work practices were measured by a 5-point scale (never, rarely, sometimes, most of the time, all the time).

#### Perceived General Health

Perceived general health was assessed with a single question “How would you rate your overall physical health?” using a 5-point scale (excellent, very good, good, fair, poor). We dichotomized the responses into excellent/very good or good/fair/poor. The self-rated health perception has been demonstrated as a good indicator of health status as well as a strong predictor of morbidity and mortality [37, 38].

#### Work-Related Injuries or Illnesses and Consequences

Respondents were asked if they had any injury or health problem at work during the past 12 months. Respondents were also asked if they had symptoms from chemicals used to perform work tasks. Questions included 16 items of acute or irritation symptoms in the respiratory, eye, skin, neurological, and gastrointestinal systems using a 5-point scale (daily, several times weekly, several times monthly, several times yearly, never in the past 12 months). For affirmative answer to work-related injury/illness or chemical-related symptoms, subsequent questions were given to indicate whether they sought medical care, if they missed work due to the symptoms, and if they reported the symptoms to their supervisor.

### Data Analysis

All study variables were described by racial and ethnic groups using descriptive statistics. Considering the distribution of respondents, race/ethnicity was categorized into three groups: Asian, Hispanic, and other (Black, White, and unknown). Chi-square tests or Fisher’s exact tests were used to compare proportions of demographic variables, general health status, work-related injuries and symptoms, and safe work behaviors among racial/ethnic groups. One-Way Analysis of Variance (ANOVA) was performed to examine differences in the means of age, job tenure, and psychosocial work factors among racial/ethnic groups. For variables presenting significant differences (p-value < 0.05), post-hoc comparisons by Tukey tests were also performed to identify specific racial/ethnic groups with significant differences. All variables significant at bivariate analysis were included in multivariable analyses. Multiple logistic regressions on health perception, chemical-related symptom reporting, and health care seeking behavior were conducted and the odds ratios (ORs) and 95% Confidence Intervals (CIs) were calculated. All analyses were carried out using the STATA version 16.0 (Stata Cooperation, College Station, TX).

## Results

### Characteristics of the Study Sample

Characteristics of the study participants are summarized in Table 1. The study sample (N = 183) consisted of 120 Asians (65.6%), 37 Hispanics (20.2%) and 27 other races (14.2%; 22 Black, 1 White, and 2 unknown); 85.7% were foreign-born and 55.7% were female. The mean age was 48 years, and the mean job tenure was 8.1 years. The proportion of foreign-born workers was higher among Asian workers than Hispanic or other workers (99.2% vs. 89.2% vs. 19.2%; p < 0.001). There were significant differences in the distribution of job location and title by racial/ethnic groups (p < 0.001). The largest proportion of Asian staff were PSAs (45.0%), and the largest proportion of Hispanic staff were campus custodians (40.5%); 5 out of 12 (41.7%) supervisors were other races. College education was less common among Asian and Hispanic workers compared to other workers (25.0% vs. 27.0% vs. 46.2%; p = 0.003).

**Table 1:** Sample characteristics of cleaning workers (N = 183)

Variable	Asian (n = 120)		Hispanic (n = 37)		Other^a^ (n = 26)		p-value^b^
	N	%	N	%	N	%	
Gender							0.215
Female	72	60.0	19	51.4	11	42.3	
Male	48	40.0	18	48.6	15	57.7	
Country of birth							**< 0.001**
United States	1	0.8	4	10.8	21	80.8	
Other	118	99.2	33	89.2	5	19.2	
Education							**0.003**
Some high school or less	43	35.8	16	43.2	1	3.8	
High school graduate	47	39.2	11	29.7	13	50.0	
College 1 year or more	30	25.0	10	27.0	12	46.2	
Job location and title							**< 0.001**
Patient support assistant, hospital	54	45.0	6	16.2	8	30.8	
Custodian, hospital	46	38.3	13	35.1	5	19.2	
Custodian, campus	16	13.3	15	40.5	8	30.8	
Supervisor	4	3.3	3	8.1	5	19.2	

	Mean	SD	Mean	SD	Mean	SD	
Age^c^ (years)	50.6	7.8	44.9	11.4	41.3	12.0	**< 0.001**
Job tenure (years)	8.5	5.8	7.9	5.4	6.7	5.1	0.321

Bold indicates significant *p* < 0.05

^a^Black (n = 22), White (n = 1), and unknown (n = 6)

^b^ANOVA, Chi-square tests, or Fisher’s exact tests were performed

^c^Among 120 Asian workers, three were excluded due to missing data

### Perception of Work Environment and Safe Work Practices

The comparison of perception of work environments and safe work practices among Asian, Hispanic, and other workers is presented in Table 2. Asian workers reported the lowest job control (p < 0.001) and the lowest supervisor support than other workers (p = 0.037), and Hispanic workers reported the lowest job demand (p = 0.016). Job strain was significantly higher among Asian workers than Hispanic Workers (p = 0.015) while the effort-reward ratios was not different between two groups. For risk perception of chemical exposure, Asian workers reported the highest score among all (p < 0.001) while there was no significant difference in perceived safety climate among the three groups. For chemical safety behaviors and PPE use, no significant differences by race/ethnicity were observed; however, for the practice of diluting concentrated products, Asian workers tended to engage less often in that safety practice than other workers (p = 0.052).

**Table 2:** Perception of work environment and safety work practices among cleaning workers (N = 183)

Variable	Asian (n = 120)		Hispanic (n = 37)		Other^a^ (n = 26)		p-value^b ^	Tuckey post-hoc test*
	Mean	SD	Mean	SD	Mean	SD		
Psychosocial factors								
Job demand (12–48)	30.8	4.5	28.8	5.8	32.0	6.9	**0.016**	H<O
Job control (24–96)	59.5	7.4	65.1	10.9	65.0	6.8	**< 0.001**	A<H, A<O
Job strain (0.125–2.0)	0.53	0.12	0.46	0.15	0.51	0.13	**0.015**	A>H
Supervisor support (4–16)	10.5	2.3	11.4	3.1	11.8	2.5	**0.037**	A<O
Coworker support (4–16)	11.8	1.5	11.6	2.4	12.3	1.6	0.368	n/a
Effort (6–30)	11.3	4.3	10.2	3.5	12.7	6.5	0.078	n/a
Reward (11–55)	49.2	5.8	48.7	6.1	47.0	10.2	0.307	n/a
Effort-reward ratio (0.11–2.73)	0.44	0.23	0.40	0.2	0.63	0.68	**0.012**	A<O, H<O
Safety climate (16–80)	56.8	10	57.8	13.8	61.3	10.6	0.205	n/a
Risk perception (1–5)	3.09	1.35	2.02	1.1	2.18	1.32	**< 0.001**	A>H, A>O

	N	%	N	%	N	%	p-value	
Chemical safety behaviors (all or most of the time)								
I follow safety rules at work	116	98.3	35	94.6	26	100	0.280	
When I use a new cleaning product, I read the label of the product	106	89.8	36	97.3	23	88.5	0.329	
I follow the directions of cleaning products	115	95.8	36	97.3	25	96.2	0.921	
I do not mix cleaning products to make them stronger	99	85.3	34	94.4	22	88.0	0.351	
I do not use concentrated products without diluting them to make them stronger	102	86.4	36	100	24	96.0	0.052	
I wash my hands before eating, drinking, or smoking	119	99.2	37	100	26	100	1.000	
When I gen chemicals on my skin, I wash my skin immediately	116	98.3	35	100	26	100	1.000	
When I use chemicals to clean an area, I ventilate the space with any available methods	93	81.6	29	78.4	21	80.8	0.912	
Personal protective equipment use (all the time)								
Glove	107	89.2	34	91.9	24	92.3	0.935	
Eye protection	17	14.2	5	13.5	6	23.1	0.491	
Face protection	11	9.2	2	5.4	1	3.6	0.77	
Surgical mask	27	22.5	9	24.3	5	19.2	0.891	
Long-sleeves or apron	24	20.0	4	10.8	8	30.8	0.144	

The sample size may vary due to missing data

*A* Asian, *H* Hispanic, *O* Other

*Significant pairs are presented (p < 0.05)

Bold indicates significant *p* < 0.05

^a^Black (n = 22), White (n = 1), and unknown (n = 6)

^b^NOVA, Chi-square tests, or Fisher’s exact tests were performed

### Perceived General Health and Work-Related Injuries or Symptoms

Table 3 presents self-rated perceived general health, work-related injuries/illnesses, chemical-related symptoms, and subsequent symptom reporting and healthcare-seeking behaviors compared by race/ethnicity. Asian workers were less likely to perceive their general health as “excellent” or “very good” than Hispanic or other workers (45.0% vs. 70.2% vs. 73.1%; p = 0.009). Asians, Hispanics, and others did not differ in the experience of work-related injuries/illnesses or chemical-related symptoms in the last 12 months. However, among the injured workers, absence from work was less common in Asian workers than in other workers (5.0% vs. 19.2%; p = 0.030). Asian workers were also less likely than Hispanic workers to report chemical-related symptoms to their supervisor (p = 0.001). Additionally, marginal significance was detected for healthcare seeking and missing work due to chemical-related symptoms; Asian workers were less likely than all other workers to visit a healthcare provider (p = 0.057) or miss work (p = 0.075).

**Table 3:** General health perception, work-related injury or illness experience and consequences among cleaning workers (N = 183)

Variable	Asian (n = 120)		Hispanic (n = 37)		Other^a^ (n = 26)		p-value^b^
	N	%	N	%	N	%	
General health							**0.009**
Excellent	12	10.0	11	29.7	6	23.1	
Very good	42	35.0	15	40.5	13	50.0	
Good	46	38.3	9	24.3	7	26.9	
Fair	19	15.8	2	5.4	0	0	
Poor	1	0.8	0	0	0	0	
Work-related injury or illness in the past year (yes)	22	18.3	6	13.2	6	23.1	0.783
Saw a health care provider (yes)	14	11.7	4	10.8	6	23.1	0.293
Missed work (yes)	6	5.0	3	8.1	5	19.2	**0.030**
Reported to the supervisor (yes)	14	11.7	5	13.5	6	23.1	0.212
Chemical-related irritation symptoms in the past year (yes)	66	55.5	24	64.9	13	48.2	0.333
Saw a health care provider (yes)	18	15.0	13	35.1	4	15.4	0.057
Missed work (yes)	6	5.0	6	16.2	3	11.5	0.075
Reported to the supervisor (yes)^c^	10	8.3	13	35.1	4	15.4	**0.001**

Bold indicates significant *p* < 0.05

^a^Black (n = 22), White (n = 1), and unknown (n = 6)

^b^Chi-square tests or Fisher’s exact tests were performed

^c^Among 66 Asian workers, two were excluded due to missing data

Table 4 summarizes the multivariable analysis results on factors associated with perceived general health status and symptom-related consequences. Significant racial and ethnic differences were observed in symptom reporting and seeking healthcare for the symptoms after controlling for age, country of birth, education, job title, job location, job strain, supervisor support, and risk perception. Compared to Asian workers, Hispanic workers were more likely to report symptoms to their supervisor (adjusted OR [aOR] = 11.67, 95% CIs 2.64–51.63) and to visit a health care provider (aOR = 14.80, 95% CIs 3.47–63.14). Other category workers were also more likely to seek health care for chemical related symptoms compared to Asian workers (aOR = 27.02, 95% CIs 1.51–482.32). For perceived general health, no significant differences were observed by race/ethnicity; however, significant associations were observed with age and education levels. Older workers were less likely to perceive their heath as excellent or very good (aOR = 0.93, 95% CIs 0.89–0.97). Workers with a college education of 1 year or more were more likely to rate their general health better (aOR = 3.20, 95% CIs 1.29–7.94) and more likely to report their chemical-related symptoms to their supervisor (aOR = 8.97, 95% CIs 1.78–45.14). As for seeking health care for chemical related symptoms, workers born in a country other than the U.S. (aOR = 26.78, 95% CIs 1.63–438.80) and working as a custodian (aOR = 4.60, 95% CIs 1.24–16.93) were more likely to seek health care than U.S.-born workers and patient support assistants, respectively.

**Table 4:** Factors associated with health perception, chemical-related symptom reporting and health care seeking among cleaning workers

Variable	Self-rated health as excellent or very good (n = 180)		Reported a chemical related symptom to the supervisor (n = 100)		Sought for health care for chemical related symptoms (n = 101)	
	OR	95% CI	OR	95% CI	OR	95% CI
Race/ethnicity (reference: Asian)						
Hispanic	2.05	0.79–5.33	**11.7**	**2.64–51.6**	**14.80**	**3.47–63.1**
Others	4.20	0.55–32.3	1.78	0.20–14.4	**27.02**	**1.51–482.3**
Age	**0.93**	**0.89–0.97**	1.03	0.97–1.09	1.05	0.99–1.12
Country of birth (Foreign born)	4.62	0.66–32.4	1.34	0.18–10.2	**26.8**	**1.63–438.8**
Education (ref. some high school or less)						
High school graduate	1.07	0.48–2.38	2.26	0.43–11.9	0.83	0.23–3.01
College 1 year or more	**3.20**	**1.29–7.94**	**8.97**	**1.78–45.1**	1.81	0.49–6.70
Job title (ref. patient support assistant)						
Custodian	1.66	0.76–3.63	0.96	0.24–3.87	**4.60**	**1.24–16.9**
Supervisor	1.19	0.25–5.53	1.33	0.09–19.2	2.13	0.19–24.1
Job location (reference: hospital)						
Campus	1.67	0.63–4.37	1.61	0.37–7.11	3.01	0.71–12.8
Job strain	0.33	0.05–2.05	1.92	0.09–40.2	0.75	0.04–12.6
Effort-reward imbalance	1.97	0.57–6.76	2.44	0.54–11.1	0.84	0.11–6.29
Supervisor support	0.95	0.80–1.12	1.18	0.92–1.54	1.00	0.78–1.28
Risk perception	0.88	0.67–1.16	1.13	0.69–1.85	1.58	0.97–2.56

*OR* odds ratio, *CI* confidence interval

Bold indicates significant *p* < 0.05

## Discussion

Occupational health disparities among immigrant and minority workers are a major concern. This study investigated racial and ethnic differences in perceptions of work environment, general health status, safe work practices, and experience of work-related injuries/illnesses among cleaning workers, which consisted of mostly racial/ethnic minority and immigrant workers. We found that occupational health disparities existed even within minority groups. Overall, Asian workers, who were largely represented by Chinese ethnicities in our sample, perceived their psychosocial work environment less favorably (e.g., lower job control, lower supervisor support, and higher job strain) than other racial/ethnic workers. Asian workers also presented more concerning behavioral patterns such as seeking less healthcare and less reporting of work-related symptoms to their supervisor.

Regarding the perception of psychosocial work environments, our findings suggest that Asian workers perceive higher job strain due to lower job control and lower supervisor support than Hispanic or other workers. Interestingly, we found that Hispanic workers perceived lower job demand and effort and subsequently, lower job strain and lower effort-reward balance compared to Asian or other workers. In a study of 237 direct care workers in nursing homes, Hurtado et al. [39] found that racial/ethnic minority workers were more likely to report high job strain and low job control than White workers. However, there are very few studies assessing racial/ethnic differences in psychosocial work environments among minority workers. Different from our findings, in a study of Las Vegas hotel room cleaners mostly consisting of Hispanics and female workers, no differences in job demand, job control, and job strain were found between Asian and Hispanic workers [40]. Previous studies have identified older, migrant, and low educated workers as a vulnerable group who experiences more stress from exposure to hazardous work conditions [41]. In our study, Asian workers had a higher proportion of older, immigrant, and low-educated workers than Hispanic and other workers. Our findings may have stemmed from such demographic characteristics of Asian workers, which put them at higher risk of psychosocial stress at work. For lower job stress observed in our study among Hispanic workers, further research is needed to elucidate the findings.

Racial/ethnic differences were not observed in general health perception after controlling for age, education, and other covariates in our study. This finding is consistent with the report of Kandula et al.’s study of 10,917 samples from California Health Interview Survey, where self-rated health (excellent/very good) was not different between Asians and Latinos [42]. Our study findings identified that older age and less education were significant risk factors for poorer health perception. This finding indicates a need for special attention to these demographic groups for health promotion.

In our study, while chemical-related symptom experience was common among workers (56% of Asian workers and 65% of Hispanic workers), Asian workers were less likely to seek health care than Hispanic or other workers. This finding is different from a previous study of hotel room cleaners that found no racial/ethnic differences in healthcare-seeking behavior [43]. The severity of symptoms may be a factor in workers seeking healthcare; those with mild symptoms may not seek care. However, differences in symptom severity by race/ethnicity is not as likely as explanation for our findings although we did not measure symptom severity. We controlled for job location and job title as proxies for chemical exposure in the multivariable analysis, and the severity of symptoms related to chemical exposures may not differ by race/ethnicity. We also note that we view race/ethnicity as sociocultural factors, not biological factors. In our study, independent of race/ethnicity, immigration status was significantly associated with seeking healthcare. Immigrant workers generally face more barriers with utilizing health care service such as financial difficulty, health insurance coverage, limited access to health care, unfamiliarity of the health care process, and limited English proficiency [44, 45]. As our study did not measure these variables, we cannot determine which, if any, of these factors played a role in seeking healthcare. Further research is needed to elucidate differences in healthcare seeking behaviors between Asian, Hispanic, and other workers.

Our study findings suggest greater underreporting of work-related symptoms among Asian workers than Hispanic or other workers. Cultural factors may play a role in behavioral responses to identify problems. Traditional Asian culture values humbleness, politeness, and deference. Collectivist tradition emphasizes conformity to these expectations and discourages emotional outburst [46]. In order to avoid shame and fears of social stigma, Asian may be unwilling to expose personal problems [46]. Cultural beliefs, coupled with well-identified barriers (e.g., fear of negative reprisals and job loss, lack of recognition and reporting mechanisms, time consuming reporting processes, and overlooking symptoms), may have influenced underreporting of work-related symptoms among Asian workers [27, 47].

Our study has several limitations. First, our relatively small convenience sample may have introduced selection bias and limited the generalizability to cleaning workers in other workplace settings. Second, the self-reported measures are effective in capturing one’s perception of work and experience of health but relying on self-reported measures may be subject to reporting bias. Social desirability or negative affectivity may have influenced the results. Although our study has the strength of including many Asian workers and foreign-born workers, our sample included only one White worker. Therefore, we could not compare minority workers groups to White workers. Additionally, the numbers of Hispanic and other racial groups as well as US-born workers were small in the sample (n = 26–37). According to Peat and Barton [48], the minimum cell size for ANOVA is 10 and in practice 20 is preferred. Although our analysis met the minimum criteria, the small cell sizes may have increased Type 1 and/or Type 2 errors. Indeed, our study observed very wide confidence intervals for race/ethnicity and country of birth variables in the multivariable analysis and, thus, had very limited precision in estimating the associations. Finally, the cross-sectional study design cannot establish causality in the observed relationships.

## New Contribution

This is one of the first studies to investigate racial/ethnic differences in various aspects including perceptions of work, general health status, healthcare seeking and work-related symptom reporting behaviors among cleaning workers. Our findings indicate that Asian workers experience a more stressful working environment and perceive poorer health status than other racial/ethnic groups and employ a passive coping reaction to work-related symptom experiences. For Hispanic workers, they are generally recognized as a vulnerable working population, but our study found that compared to Asian workers, they reported relatively better general health perception and active coping reaction to work-related symptom experiences. Additionally, Hispanic workers presented the lowest risk perception and effort-reward imbalance ratio. Nonetheless, the majority of Hispanic workers as well as Asian workers were shown to experience symptoms related to chemical exposure at work. Management support, empowerment, and training for workers, especially minority workers such as Asian and Hispanic workers, would help improve their work environment and health. Future research with a larger sample representing more diverse racial/ethnic groups is needed to validate our study findings.
